# Prognostic models for survival and consciousness in patients with primary brainstem hemorrhage

**DOI:** 10.3389/fneur.2023.1126585

**Published:** 2023-02-23

**Authors:** Jingyi Zhou, Rui Wang, Jizhong Mao, Yichen Gu, Anwen Shao, Fengqiang Liu, Jianmin Zhang

**Affiliations:** ^1^Department of Neurosurgery, Second Affiliated Hospital, School of Medicine, Zhejiang University, Hangzhou, Zhejiang, China; ^2^Liangzhu Laboratory, Zhejiang University Medical Center, Hangzhou, Zhejiang, China; ^3^Brain Research Institute, Zhejiang University, Hangzhou, Zhejiang, China; ^4^Collaborative Innovation Center for Brain Science, Zhejiang University, Hangzhou, Zhejiang, China; ^5^Stroke Research Center for Diagnostic and Therapeutic Technologies of Zhejiang Province, Hangzhou, China; ^6^Clinical Research Center for Neurological Diseases of Zhejiang Province, Hangzhou, China

**Keywords:** primary brainstem hemorrhage, consciousness, multiple logistic regression, predictive factors, stereotactic aspiration

## Abstract

**Objectives:**

Primary brainstem hemorrhage (PBSH) is one of the most catastrophic spontaneous intracerebral hemorrhage diseases, with a mortality rate of 70–80%. We explored the predictive factors for survival and consciousness in patients with PBSH (ClinicalTrials.gov ID: NCT04910490).

**Methods:**

We retrospectively reviewed 211 patients with PBSH admitted to our institution between January 2014 and October 2020. Clinical outcomes included the 30-day survival rate and the 90-day consciousness rate as evaluated by the National Institutes of Health Stroke Scale score. Multiple logistic regression analysis was performed.

**Results:**

The overall 30-day survival rate of 211 patients with PBSH was 70%. Several predictive factors including hematoma volume, hematoma location, activated partial thromboplastin time (APTT) upon admission, and therapeutic strategy were significantly related to 30-day survival. Compared with conservative treatment, stereotactic aspiration in our prediction model is strongly associated with improved 30-day survival (odds ratio, 6.67; 95% confidence interval, 3.13–14.29; *P* < 0.001). The prognosis prediction model of 90-day consciousness including factors such as mydriasis, APTT value, hematoma location, and hematoma volume upon admission has a good predictive effect (AUC, 0.835; 95% confidence interval, 0.78–0.89; *P* < 0.001).

**Conclusion:**

In patients with PBSH, conscious state upon admission, coagulation function, hematoma volume, hematoma location, and therapeutic strategy were significantly associated with prognosis. Stereotactic aspiration could significantly reduce the 30-day mortality rate.

## 1. Introduction

Primary brainstem hemorrhage (PBSH) is one of the most catastrophic spontaneous intracerebral hemorrhage (ICH) diseases, which accounts for approximately 3.8–6.3% of ICH cases (1). PBSH is known to have a poor prognosis, with a mortality rate varying widely from 70 to 80% (2, 3), and the 30-day mortality rate for patients with a Glasgow Coma Scale (GCS) score of < 5 and a hematoma volume of >10 ml even reaches 100% (4). Previous studies have found that the severity of initial neurological symptoms, hydrocephalus, hematoma volume, and GCS score may be predictors of poor outcomes for patients with PBSH (5, 6), but the conclusions of different studies are not uniform. Currently, there is no standard, widely accepted prognostic model or clinical grading scale for survival and consciousness outcomes, and there are no strong recommendations for clinical treatment strategies for PBSH.

Neurosurgical interventions continue to be controversial and have not been sufficiently investigated because ICH in the posterior fossa has been excluded from large ICH surgical intervention trials in the past (7, 8). The American Heart Association/American Stroke Association Guidelines clearly advise against the surgical evacuation of brainstem hematoma (9). However, others have advocated the efficacy of surgical treatment for PBSH or other brainstem diseases (10, 11). The number of patients with PBSH who underwent surgery enrolled in previous studies has been relatively small, and most studies date back to more than 20 years. Computed tomography (CT)-guided stereotactic hematoma puncture and drainage provided a potential treatment for PBSH, which showed a more favorable outcome than conservative therapy (12, 13). The option of PBSH treatments including conservative treatment or stereotactic surgery remains quite challenging for neurosurgeons. Accordingly, studies on PBSH treatments related to death or a better outcome are useful in clinical practice.

Here, we conducted a retrospective, observational, explorative, and single-center study to analyze the prognostic factors affecting the 30-day survival rate and the 90-day consciousness rate in 211 patients with PBSH undergoing conservative or stereotactic aspiration therapy. The present study aimed to identify chief predictors of survival and consciousness after PBSH.

## 2. Materials and methods

### 2.1. Study design

Using a retrospective, observational, explorative, and single-center study design, we aimed to explore the prognostic factors for survival and consciousness in patients with PBSH. This study was conducted with the approval of the Ethics Committee of the Second Affiliated Hospital Zhejiang University School of Medicine. Due to the retrospective, observational nature of this study and the anonymity of patients, the need for informed consent was waived. The objectives of this study were fully explained to patients or patients' families during follow-up. Moreover, this study tried to present the results of an explorative analysis without an *a priori* hypothesis for the prognostic factors.

### 2.2. Patient selection

We retrospectively reviewed the data of 211 patients with PBSH from 342 consecutive patients admitted to our institution between January 2014 and October 2020. The inclusion criteria were as follows: (1) a diagnosis of PBSH confirmed by CT and (2) complete clinical data (laboratory data, imaging data, and other clinical data). The exclusion criteria were as follows: (1) secondary brainstem hemorrhage caused by trauma, thrombolytic therapy, cavernous hemangioma, or arteriovenous malformation, (2) surgical treatment of brainstem hemorrhage before admission to our hospital, (3) admission to our hospital more than 10 days after symptom onset, and (4) missed follow-up.

### 2.3. Clinical data

All patients' clinical data were reviewed including general characteristics (age, sex, smoking or drinking habits, previous functional status, and comorbidities), clinical characteristics upon admission (vital signs, blood pressure, pupillary abnormalities, GCS score, and emergency treatment), laboratory data, radiological findings upon admission or during hospitalization, treatment, and outcomes. Clinical data during hospitalization referred to the examination data from admission to hospital discharge when people have recovered sufficiently or can be appropriately rehabilitated elsewhere or died, except for the first data upon admission.

**Figure 1:**
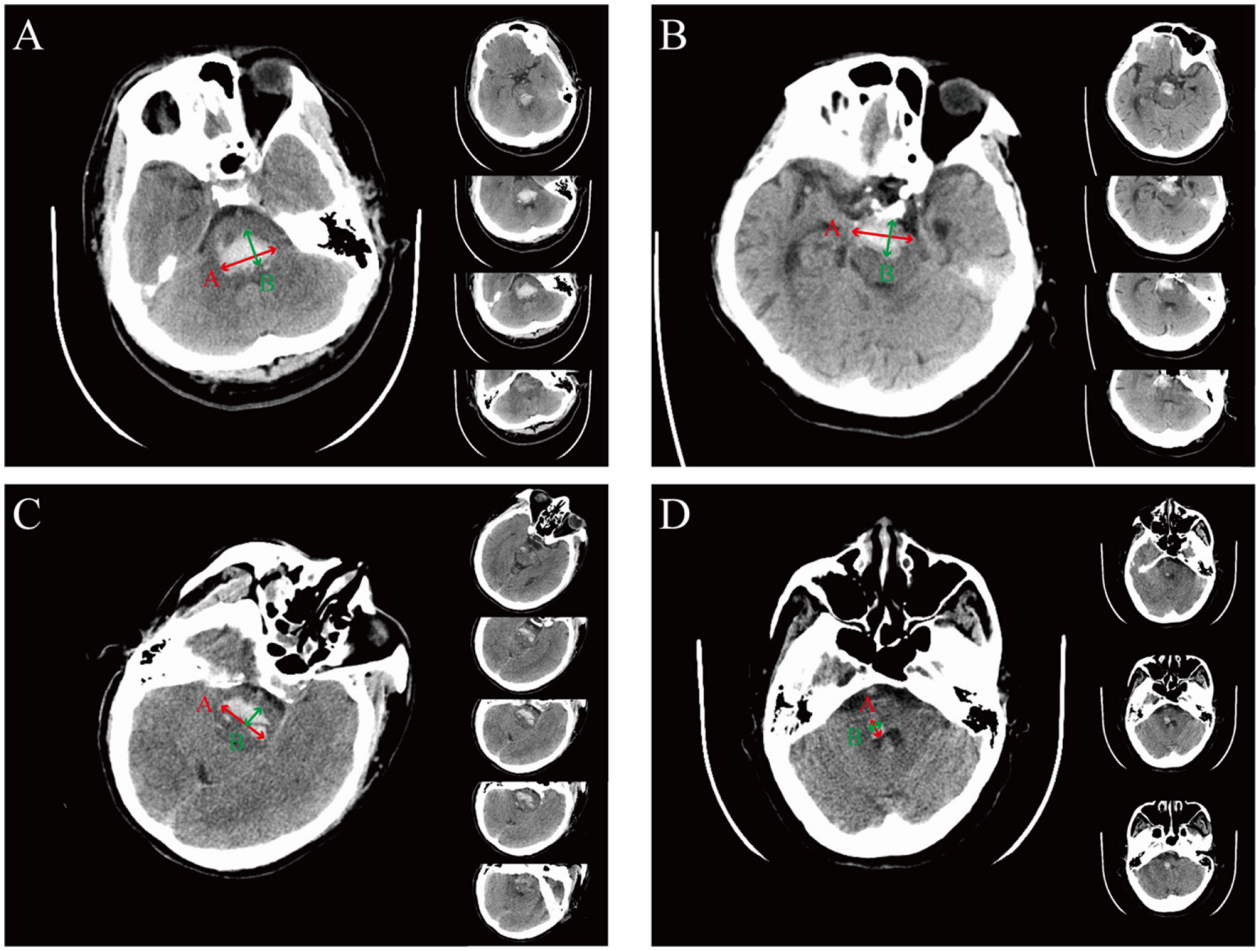
The representation of the ABC formula for calculating hemorrhage volume in different hemorrhagic locations. **(A)** Representative images of dorsal hematoma. The A value is defined as the longest segment (red arrow) in the largest hematoma slice, the B value is defined as the longest segment, which is perpendicular to the A segment on the same slice (green arrow), and the C value is defined as the approximate number of CT slices (right panel) with the slice thickness (being 0.5 cm in this example). For this example, (A × B × C)/2 = [3.2 × 1.9 × (4 × 0.5)]/2 = 6.08 cm^3^. **(B)** Representative images of ventral hematoma. For this example, (A × B × C)/2 = [3.1 × 1.5 × (4 × 0.5)]/2 = 4.65 cm^3^. **(C)** Representative images of hematoma crossing the midline. For this example, (A × B × C)/2 = [3.1 × 1.1 × (5 × 0.5)]/2 = 4.26 cm^3^. **(D)** Representative images of hematoma without crossing the midline. For this example, (A × B × C)/2 = [0.9 × 0.7 × (3 × 0.5)]/2 = 0.47 cm^3^.

Upon admission to the emergency department, head CT plain scans were performed to assess the severity of the disease. The features evaluated on CT included the location and the extension of hemorrhage, hematoma volume, and the presence of hydrocephalus. Lesions located entirely within the cerebellum, the thalamus, the basal ganglia, or the ventricle were excluded, but lesions extending into these regions from the brainstem were included. Hemorrhage volume is calculated as follows: volume = (A × B × C)/2 where A is the greatest hemorrhage diameter by CT, B is the diameter perpendicular to A, and C is the approximate number of CT slices with hemorrhage multiplied by the slice thickness (14), as shown in Figure 1. Hydrocephalus on CT was determined by enlarged ventricles or obstruction to the flow of cerebrospinal fluid within the ventricular system. Clinical data were reviewed, and radiological data were assessed by two trained neurosurgeons blinded to outcome.

### 2.4. Stereotactic aspiration treatment

The selection criteria for surgery were as follows: hematoma volume >5 ml and GCS <8. All patients included in the study, who meet the selection criteria mentioned earlier and have no contraindications of surgery such as severe disorders of blood coagulation, were offered the option of surgery. Their relatives either agreed to surgical intervention or refused. Patients whose families consented to the surgery were allocated to the surgery group, and those patients whose families refused the surgery were put into the conservative treatment group.

### 2.5. Assessments of outcome

The 30-day survival rate and 90-day consciousness rate were chosen as the primary outcomes. Mortality data were obtained from medical records or by telephone contact with primary care physicians or family members. Consciousness was determined based on the National Institutes of Health Stroke Scale (NIHSS) (item 1a: value 0, 1, or 2 for consciousness, value 3 for unconsciousness), which was obtained from a clinic visit at 90-day follow-up or by telephone contact by two trained neurosurgeons blinded to research data.

### 2.6. Statistical analysis

R (version 4.0.3) and RStudio (version 1.4.1106) were used for statistical calculations. Normally distributed continuous variables are presented as mean ± standard deviation (SD), and non-normally distributed continuous variables are presented as median with interquartile range (IQR). Normally distributed continuous variables were compared by the two-sided *t*-test, and non-normally distributed continuous variables and ordinal variables were compared by the Mann–Whitney–Wilcox test. Categorical variables were compared by the Pearson chi-square test, the continuity-corrected chi-square test, or Fisher's exact test (expected frequency <5). Odds ratios (ORs) and 95% confidence intervals (CIs) were also reported.

Univariate analysis was used initially to identify possible relations between outcomes. Variables were only modeled using multiple logistic regression if they were statistically significant and biologically related to clinical outcomes. A pre-treatment prognosis prediction model including only admission indicators and a post-treatment prognosis prediction model including both admission and hospitalization indicators were established. The predictive effect was evaluated by the receiver operating curve (ROC) analysis. For the area under the ROC curve (AUC), a value of >0.8 or 0.7–0.8 represents an excellent or good prediction ability, respectively. The *p*-values of <0.05 were regarded as statistically significant.

## 3. Results

### 3.1. Baseline characteristics

**Figure 2:**
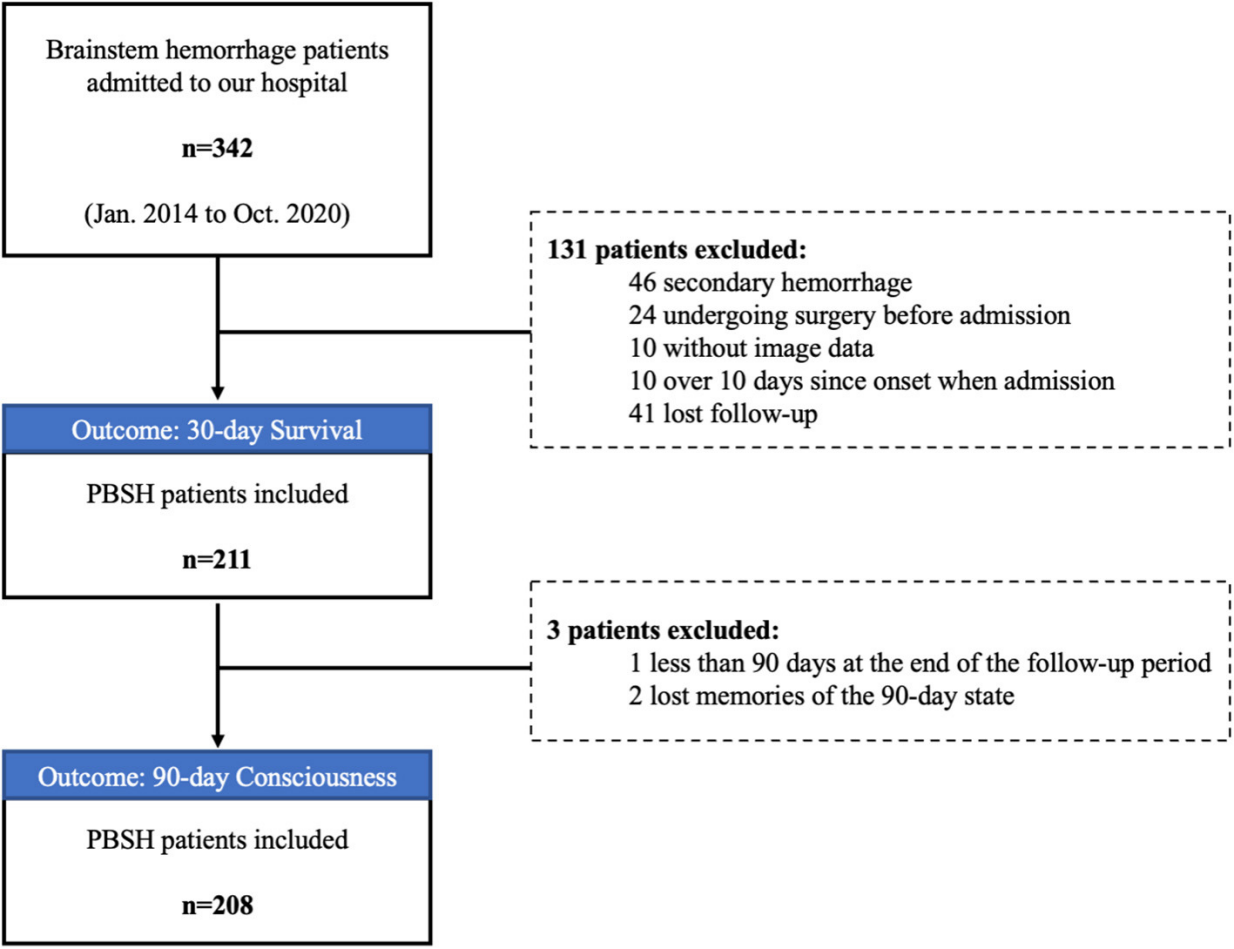
Flow chart of patient enrollment.

A total of 211 patients with PBSH were included in the final analysis (Figure 2). Table 1 shows the general characteristics of included patients in the baseline and the variance data according to survival outcomes. The median age of the 211 patients was 49 years. Of the 175 (83%) male and 36 (17%) female patients, 153 (73%) had a history of hypertension, 26 (12%) had diabetes, and 21 (10%) had a history of stroke or myocardial infarction. The mean body mass index was 24.9 ± 4.4, suggesting a trend toward being overweight.

**Table 1:** General characteristics of study population.

**Characteristic**	**211 patients**	**30-day survival group (*n* = 147)**	**30-day death group (*n* = 64)**	***P* value**
Age (years)	49 (42–57)	50 (42–57)	48 (42–57)	0.462
Male	175 (83%)	119 (81%)	56 (88%)	0.335
Hypertension	153 (73%)	111 (76%)	42 (66%)	0.162
Diabetes mellitus	26 (12%)	19 (13%)	7 (11%)	0.860
History of stroke or myocardial infarction	21 (10%)	15 (10%)	6 (9%)	1
Smoking habits (pack-years)	0 (0–14)	0 (0–11)	0 (0–15)	0.816
Drinking habits (alcohol use)	0 (0–0)	0 (0–0)	0 (0–8)	0.311
Hyperuricemia	6 (3%)	3 (2%)	3 (5%)	0.540
Anticoagulant drugs	8 (4%)	5 (3%)	3 (5%)	0.954
Antiplatelet drugs	9 (3%)	9 (6%)	0 (0%)	0.097
Lipid-lowering drugs	5 (3%)	3 (2%)	2 (3%)	0.634
Body mass index	24.9 ± 4.4	24.9 ± 3.7	24.9 ± 5.7	0.997

Data are expressed as n (%), mean ± SD, median (IQR), as appropriate.

Body mass index = weight/height^2^ (kg/m^2^).

Pack-years of cigarette smoking = Packs per day (one pack contains 20 cigarettes) × Years.

Alcohol use = Drink units of alcohol per week (one unit contains 12 grams of alcohol).

The mean blood pressure upon admission was 166/95 ± 31/19 mmHg (Table 2), which was consistent with previous reports that hypertension was the most common risk factor of PBSH (15, 16). The median GCS score upon admission was 4 (IQR, 3–5), and 90% of patients were in a coma upon admission.

**Table 2:** Associations of characteristics upon admission with 30-day survival.

**Characteristic**	**All patients (*n* = 211)**	**Survivor (*n* = 147)**	**Non-survivor (*n* = 64)**	***P* value**	**OR (95% CI)**
**Clinical characteristics upon admission**					
Heart rate	84 (71–101)	82 (71–98)	89 (70–112)	0.149	0.99 (0.98–1.01)
Respiratory rate	12 (4–15)	12 (3–15)	12 (4–15)	0.398	1.02 (0.97–1.06)
Systolic blood pressure (mmHg)	166 ± 31	164 ± 29	170 ± 35	0.211	1.00 (0.99–1.02)
Diastolic blood pressure (mmHg)	95 ± 19	93 ± 18	99 ± 22	0.069	0.98 (0.96–1.01)
Anisocoria	57 (27%)	37 (25%)	20 (31%)	0.456	0.74 (0.39–1.41)
Mydriasis	37 (18%)	18 (12%)	19 (30%)	0.004^**^	0.33 (0.16–0.68)
Pinpoint pupils	57 (27%)	32 (22%)	25 (39%)	0.015^*^	0.43 (0.23–0.82)
Abnormal light reflex	182 (86%)	122 (83%)	60 (94%)	0.075	0.33 (0.11–0.98)
GCS score	4 (3–5)	4 (3–5)	3 (3–5)	0.099	1.14 (1.00–1.29)
Emergency hemostatic drugs	78 (37%)	60 (41%)	18 (28%)	0.129	1.75 (0.93–3.33)
Emergency mechanical ventilation	190 (90%)	131 (89%)	59 (92%)	0.664	0.69 (0.24–2.00)
Emergency decompression	123 (58%)	86 (59%)	37 (58%)	1	1.03 (0.57–1.85)
Emergency external ventricular drainage	13 (6%)	10 (7%)	3 (5%)	0.783	1.49 (0.39–5.56)
**Image characteristics upon admission**					
Hematoma volume (ml)	9.6 (5.8–14.8)	9.2 (5.3–14.0)	11.8 (7.7–17.7)	0.006^**^	0.94 (0.91–0.98)
**Location of hemorrhage**					
Dorsal location	99 (47%)	60 (41%)	39 (61%)	0.011^*^	0.44 (0.24–0.81)
Crossing the midline	190 (90%)	128 (87%)	62 (97%)	0.053	0.22 (0.05–0.96)
Midbrain	151 (72%)	106 (72%)	45 (70%)	0.920	1.09 (0.57–2.08)
Pons	207 (98%)	143 (97%)	64 (100%)	0.434	*NA*
Medulla	12 (6%)	7 (5%)	5 (8%)	0.578	0.58 (0.18–1.92)
**Extension of hemorrhage**					
Cerebellum	34 (16%)	20 (14%)	14 (22%)	0.194	0.56 (0.26–1.20)
Thalamus	24 (11%)	12 (8%)	12 (10%)	0.047^*^	0.38 (0.16–0.91)
Basal ganglia	23 (11%)	13 (9%)	10 (16%)	0.225	0.52 (0.22–1.27)
Ventricle	125 (59%)	82 (56%)	43 (67%)	0.162	0.62 (0.33–1.14)
Hydrocephalus	8 (4%)	4 (3%)	4 (6%)	0.399	0.42 (0.10–1.72)
**Laboratory blood examinations upon admission**					
White blood cell count (10^12^/L)	11.3 (9.0–13.7)	11.0 (8.8–13.5)	11.8 (9.5–15.7)	0.093	0.93 (0.87–1.00)
Hemoglobin (g/L)	133.2 ± 20.6	131.3 ± 16.7	137.6 ± 27.0	0.093	0.99 (0.97–1.00)
Platelet (10^9^/L)	179.8 ± 60.4	184.8 ± 57.0	168.4 ± 66.4	0.094	1.01 (1.00–1.01)
C-reactive protein (mg/L)	31 (10–77)	33 (13–76)	24 (5–75)	0.199	1.00 (1.00–1.00)
Blood glucose (mmol/L)	7.6 (6.5–8.9)	7.5 (6.4–8.8)	8.0 (6.9–9.3)	0.239	1.00 (0.94–1.07)
Blood creatinine (μmol/L)	76 (62–108)	73 (58–96)	91 (70–134)	<0.001^***^	0.99 (0.98–0.99)
TnT (ng/ml)	0.02 (0.01–0.05)	0.02 (0.01–0.04)	0.04 (0.02–0.18)	<0.001^***^	0.13 (0.03–0.52)
APTT (s)	36.3 ± 6.0	35.7 ± 5.4	37.7 ± 7.1	0.045^*^	0.95 (0.90–0.99)
D-Dimer (μg/L)	2,045 (882–4,962)	1,860 (860–3,990)	2,410 (890–5,080)	0.492	1.00 (1.00–1.00)

^*^P < 0.05, ^**^P < 0.01, ^***^P < 0.001.

Data are expressed as n (%), mean ± SD, median (IQR), OR (95% CI), as appropriate.

TnT, troponin T; APTT, activated partial thromboplastin time.

NA, not available.

The mean hematoma volume on admission CT scans was 11.3 ± 7.9 ml, and the median volume was 9.6 (IQR, 5.8–14.8) ml. A hematoma crossing the midline of the brainstem was found in 190 (90%) patients, and a dorsal location was found in 99 (47%) patients. Intraventricular hemorrhage was found in 125 (59%) patients, and hydrocephalus was observed in eight (4%) patients on emergency CT.

The median white blood cell count upon admission was 11.3 (IQR, 9.0–13.7) × 10^12^ cells/L, and the median C-reactive protein level was 31 (IQR, 10–77) mg/L, indicating that inflammation may have appeared in the early course of PBSH.

### 3.2. Factors upon admission and during hospitalization influencing 30-day survival

The overall 30-day survival rate of 211 patients was 70%. The 30-day survival outcome was significantly related to the following factors upon admission (Table 2): mydriasis (*P* = 0.004; OR, 0.33; 95% CI, 0.16–0.68), pinpoint pupils (*P* = 0.015; OR, 0.43; 95% CI, 0.23–0.82), hematoma volume (*P* = 0.006; OR, 0.94; 95% CI, 0.91–0.98), dorsal brainstem hematoma (*P* = 0.011; OR, 0.44; 95% CI, 0.24–0.81), extension into the thalamus (*P* = 0.047; OR, 0.38; 95% CI, 0.16–0.91), blood creatinine (*P* < 0.001; OR, 0.99; 95% CI, 0.98–0.99), troponin T (TnT, *P* < 0.001; OR, 0.13; 95% CI, 0.03–0.52), and activated partial thromboplastin time (APTT, *P* = 0. 045; OR, 0.95; 95% CI, 0.90–0.99).

Among all 211 patients, 113 patients underwent stereotactic aspiration treatment and 98 patients underwent conservative treatment (Supplementary Table S1). Univariate analysis showed that stereotactic aspiration treatment (*P* < 0.001; OR, 3.13; 95% CI, 1.67–5.88) and changes in blood sodium levels (*P* < 0.01) during hospitalization were significantly related to 30-day survival.

**Figure 3:**
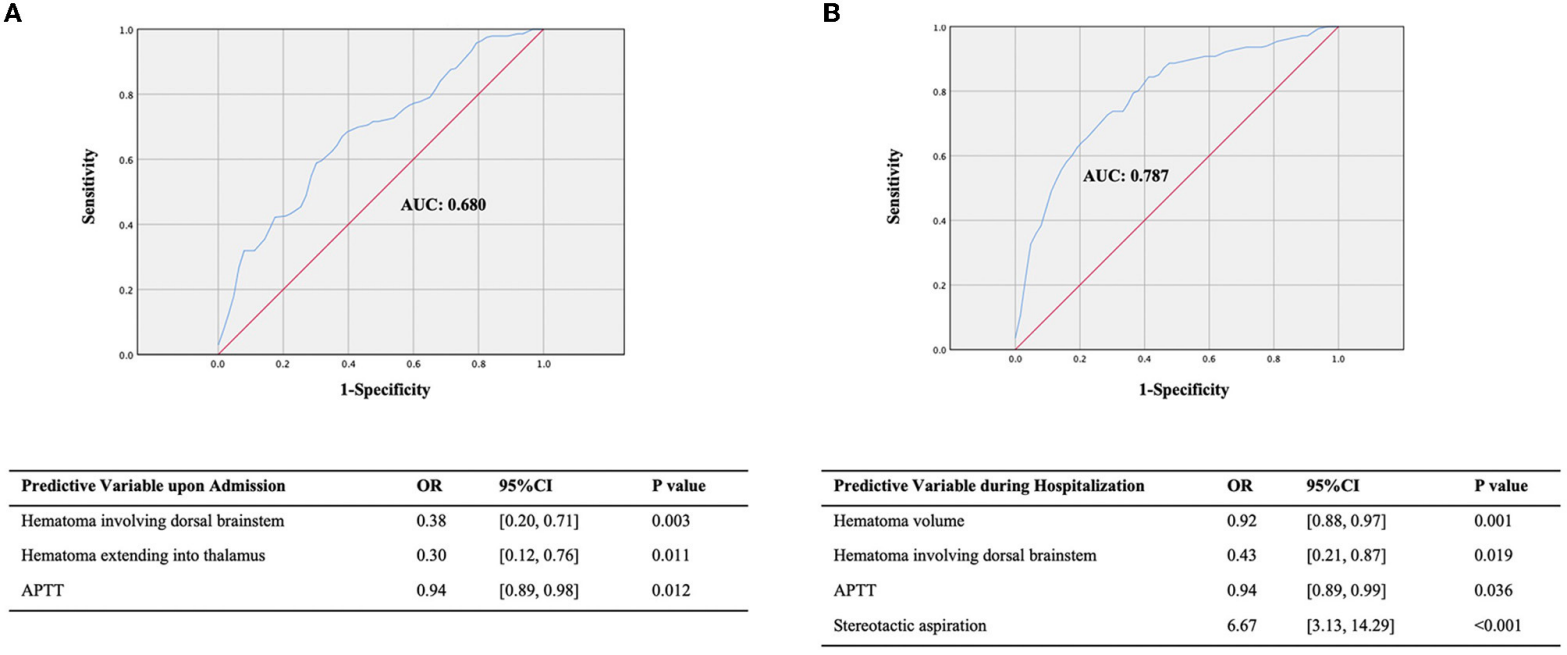
ROC of the prediction model for the 30-day survival outcome. **(A)** The prediction model for the 30-day survival outcome upon admission. **(B)** The prediction model for the 30-day survival outcome during hospitalization.

We selected clinically related factors from the significant factors affecting 30-day survival upon admission and during hospitalization to construct a prognostic prediction model. In total, eight factors upon admission were found to be significantly associated with 30-day survival; of which, three factors were selected to build the pre-treatment prognosis prediction model: dorsal brainstem hematoma, showing a close relationship with survival outcome in previous reports (16, 17); extension into the thalamus, suggesting a vertically widespread extension of the hematoma; and APTT, reflecting the state of endogenous coagulation in the body. The post-treatment model incorporated four factors both upon admission and during hospitalization, including hematoma volume, dorsal brainstem hematoma, APTT, and treatment method. The pre-treatment model showed a significant predictive effect (AUC, 0.680; 95% CI, 0.60–0.76; *P* < 0.001; Figure 3A). The post-treatment model, which incorporated the treatment method as a factor, had a better predictive effect (AUC, 0.787; 95% CI, 0.71–0.85; *P* < 0.001; Figure 3B). Compared with conservative treatment, stereotactic aspiration is strongly associated with 30-day survival (OR, 6.67; 95% CI, 3.13–14.29; *P* < 0.001; Figure 3B). Larger admission hematoma volume (OR, 0.92; 95% CI: 0.88–0.97; *P* = 0.001), higher APTT (OR, 0.94; 95% CI: 0.89–0.99; *P* = 0.036), and dorsal brainstem hematoma (OR, 0.43; 95% CI: 0.21–0.87; *P* = 0.019) upon admission are risk factors for 30-day death in the post-treatment model.

### 3.3. Factors upon admission and during hospitalization influencing 90-day consciousness

Among the 211 patients included in the present study, one patient had not yet reached 90 days at the end of the follow-up period, and two patients had lost their memory of the 90-day state. Therefore, we performed statistical analysis on the data of the remaining 208 patients. Univariate analysis of baseline data upon admission revealed that mydriasis (*P* = 0.017; OR, 0.29; 95% CI, 0.11–0.78), abnormal light reflex (*P* = 0.002; OR, 0.25; 95% CI, 0.11–0.57), GCS score (*P* < 0.001; OR, 1.40; 95% CI, 1.22–1.61), emergency mechanical ventilation (*P* = 0.020; OR, 0.31; 95% CI, 0.12–0.78), hematoma volume (*P* < 0.001; OR, 0.85; 95% CI, 0.80–0.91), dorsal location (*P* < 0.001; OR, 0.27; 95% CI, 0.14–0.51), hematoma across the midline (*P* = 0.011; OR, 0.28; 95% CI, 0.11–0.71), hematoma expanding to the thalamus (*P* = 0.028; OR, 0.27; 95% CI, 0.08–0.93), extension into the basal ganglia (*P* = 0.037; OR, 0.28; 95% CI, 0.08–0.99), the presence of intraventricular hemorrhage (*P* < 0.001; OR, 0.34; 95% CI, 0.18–0.61), hydrocephalus signs (*P* = 0.047), TnT (*P* < 0.001; OR, 0.00; 95% CI, 0.00–0.01), and APTT (*P* < 0.001; OR, 0.91; 95% CI, 0.85–0.97) upon admission were significantly associated with the 90-day consciousness outcome (Supplementary Table S2). Factors during hospitalization including high fever (*P* = 0.002; OR, 0.46; 95% CI, 0.25–0.84), myocardial injury (*P* = 0.029; OR, 0.31; 95% CI, 0.10–0.93), and blood potassium reduction (*P* = 0.042; OR, 1.66; 95% CI, 1.01–2.71) were significantly associated with the 90-day consciousness outcome (Table 3).

**Table 3:** Associations of characteristics during hospitalization with 90-day consciousness.

**Characteristic**	**All patients (*n* = 208)**	**Conscious patients (*n* = 67)**	**Unconscious patients (*n* = 141)**	***P* value**	**OR (95% CI)**
**Laboratory blood examinations during hospitalization**					
Blood creatinine increase (μmol/L)	8 (−2 to 33)	4 (−2 to 18)	10 (−1 to 44)	0.156	1.00 (0.99 to 1.00)
Blood sodium increase (μmol/L)	4.8 (1.0 to 10.7)	4.2 (0.6 to 8.5)	5.1 (1.1 to 11.8)	0.097	0.96 (0.93 to 1.00)
Blood sodium decrease (μmol/L)	−7.0 (−12.0 to−2.9)	−7.0 (−10.0 to−4.0)	−7.4 (−13.1 to −2.3)	0.518	1.01 (0.97 to 1.05)
Blood potassium increase (μmol/L)	0.9 (0.4 to 1.4)	0.8 (0.4 to 1.2)	0.9 (0.4 to 1.4)	0.359	0.71 (0.48 to 1.06)
Blood potassium decrease (μmol/L)	−0.4 (−0.9 to −0.1)	−0.3 (−0.7 to −0.1)	−0.4 (−1.0 to −0.1)	0.042^*^	1.66 (1.01 to 2.71)
**Treatment method**					
Stereotactic aspiration	112 (54%)	35 (52%)	77 (55%)	0.864	0.91 (0.51 to 1.64)
**Complications during hospitalization**					
High fever	137 (66%)	36 (54%)	101 (72%)	0.002^**^	0.46 (0.25 to 0.84)
Intracranial infection	3 (1%)	0 (0%)	3 (2%)	0.229	*NA*
Pneumonia	182 (88%)	59 (88%)	123 (87%)	0.470	1.08 (0.44 to 2.63)
Other infections	22 (11%)	3 (4%)	19 (13%)	0.049^*^	0.30 (0.09 to 1.05)
Deep venous thrombosis	12 (6%)	4 (6%)	8 (6%)	0.932	1.05 (0.31 to 3.57)
Myocardial injury	28 (14%)	4 (6%)	24 (17%)	0.029^*^	0.31 (0.10 to 0.93)
Bed sores	30 (14%)	10 (15%)	20 (14%)	1	1.06 (0.46 to 2.44)
Secondary epilepsy	10 (5%)	1 (1%)	9 (6%)	0.123	0.22 (0.03 to 1.79)
Digestive tract hemorrhage	3 (1%)	1 (1%)	2 (1%)	0.967	1.05 (0.09 to 11.11)

^*^P < 0.05, ^**^P < 0.01.

Data are expressed as n (%), mean ± SD, median (IQR), OR (95% CI), as appropriate.

NA, not available.

**Figure 4:**
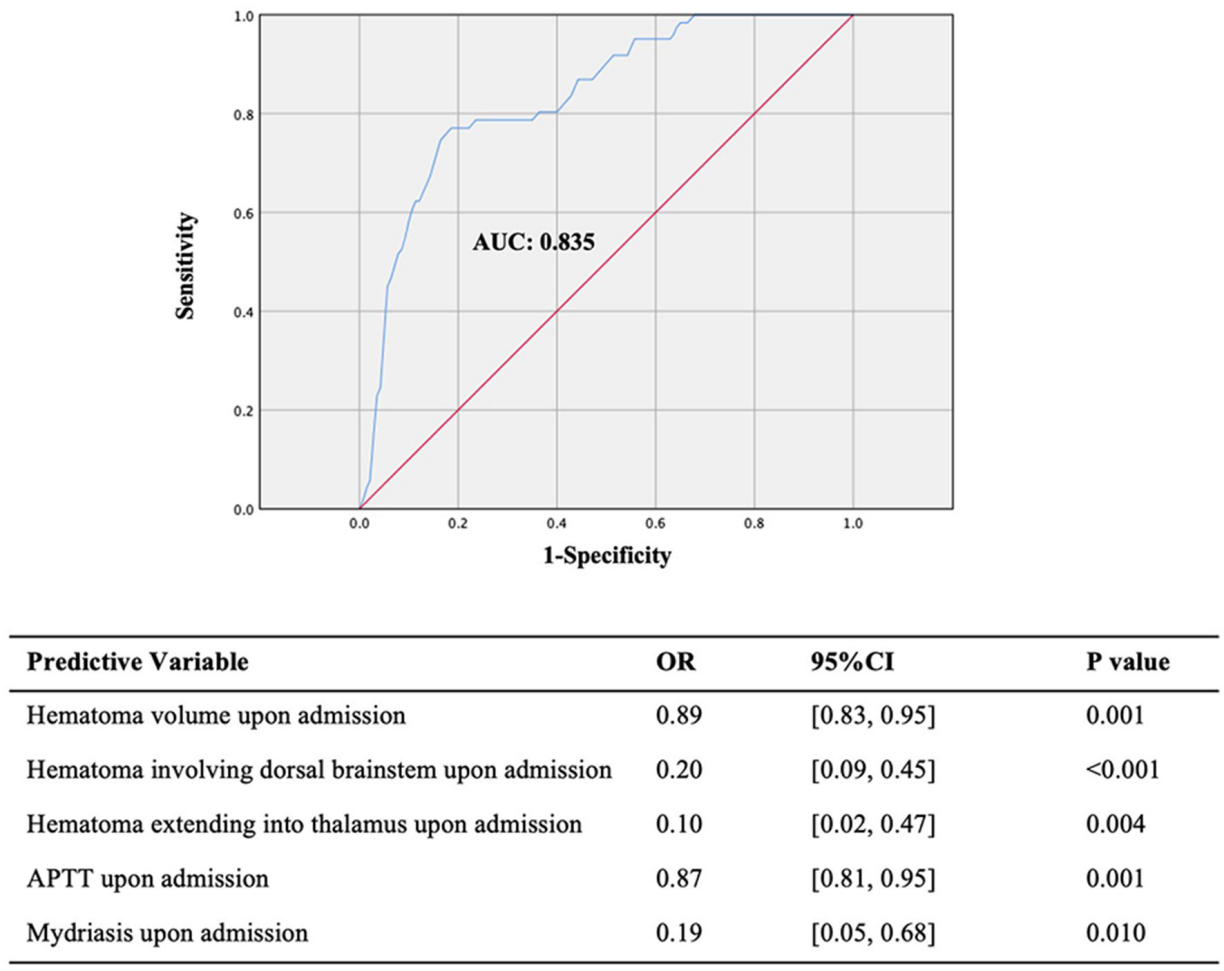
ROC of the prediction model for the 90-day consciousness outcome.

As before, multiple logistic regression analysis was performed to construct a prognostic prediction model. We found that the prognosis prediction model upon admission was consistent with that during hospitalization, indicating no more risk factors during hospitalization for the 90-day consciousness outcome. According to our statistical analysis and potential biological links of the significant predictive factors with treatment outcome, we selected five factors out of 19 significant factors to build the prognosis prediction model: hematoma volume, dorsal brainstem hematoma, and extension into the thalamus, which may be associated with the impaired reticular activating system and its projections posteriorly to the thalamus; APTT, implying the possibility of rebleeding and imperceptible microbleeds during hospitalization; and mydriasis, involving the oculomotor nerve or oculomotor nuclei in the deep brainstem. The 90-day consciousness prognosis prediction model had an excellent predictive effect (AUC, 0.835; 95% CI, 0.78–0.89; *P* < 0.001; Figure 4). Dorsal brainstem and thalamus involvement, higher APTT, and mydriasis upon admission are risk factors for 90-day unconsciousness in the prediction model.

## 4. Discussion

In the present study, we found that several factors are positively or negatively associated with 30-day survival and 90-day consciousness outcome, including clinical variables, radiological variables, and laboratory variables upon admission and during hospitalization, in a large database of 211 patients with PBSH.

### 4.1. Epidemiology

The incidence of ICH is higher among Chinese and men than among Caucasians and women, respectively (18). It is noteworthy that there is a prominent prevalence of male sex in the population with PBSH. For example, 202 (72%) out of 281 subjects and 59 (79%) out of 75 subjects were male in Korean and Chinese study populations, respectively (19, 20). In accordance with these results, the present study also showed a higher prevalence of men (83%). The reasons underlying the higher incidence of ICH and the higher risk of PBSH in men in the Chinese population are not clear but may be related to differences in the prevalence of vascular risk factors, personal living habits, and health conditions prior to their illness. Chinese people have been found to have higher rates of hypertension and higher susceptibility to micro-bleeding events and intracranial hemorrhage due to anticoagulation medication compared with other ethnic groups, which are more common in men (21, 22). Although men had a higher incidence, gender was not a prognostic factor in this study. A possible explanation could be that if a brainstem hemorrhage occurred, the clinical outcome does not differ significantly because of its high rate of mortality.

### 4.2. Radiological factors associated with primary outcomes

#### 4.2.1. Hematoma location

There is currently no unified classification for PBSH. In a study of 62 cases published in 1992, a close relationship was observed between overall survival and hematoma location, in which case, the survival rate is the highest in the small unilateral tegmental type and the lowest in the massive type with its bilateral spread into both the basis pontis and the tegmentum (21). Studies using another classification system emphasized the importance of the ventral and dorsal sides and reported that dorsal hematoma showed better recovery than ventral hematoma (6, 16). However, in the present study, the dorsal location of the hematoma is related to an unfavorable outcome. This might be explained by the fact that hematoma of the dorsal type is caused by rupture of the penetrating and long circumferential vessels that enter the tegmentum dorsally and course medially, resulting in rapid destruction of the brain stem as well as the reticular activating system in the pontine tegmentum.

#### 4.2.2. Hematoma extension associated with primary outcomes

Hematoma extension into the midbrain with or without the thalamus was shown to be independently associated with death (23, 24). Here, thalamic involvement could predict adverse outcomes, including death and unconsciousness, which might be caused by a vertically widespread extension of brainstem hematoma. Moreover, intraventricular extension and acute hydrocephalus were not correlated with death but were significantly more common in unconscious patients who survived, indicating a non-lethal but impaired functional outcome. The possible explanation may be that the primary brain injury to the vital structures within the brain parenchyma is more important than the secondary injury consisting of mass effect and toxic effect in the ventricular system, causing different clinical outcomes in the short and long term.

### 4.3. Laboratory evaluation

Abnormal coagulation function implies the possibility of rebleeding where APTT is the main indicator of endogenous coagulation status *in vivo* and it changes with coagulation status. Previous studies have found it to be an independent risk factor for cerebral micro-bleeding events in patients with ICH (25). Similarly, we found higher APTT was a significant predictor of death and unconsciousness in patients with PBSH. Therefore, for PBSH with prolonged APTT, further imaging examination should be advised for screening micro-bleeding events, which can further guide the treatment assessment.

### 4.4. Surgical management and its impact on outcomes

Neurosurgical interventions such as hematoma evacuation remain controversial for PBSH because randomized clinical studies have failed to demonstrate a clear benefit to surgical management. The efficacy and safety of surgery for patients with PBSH remain debatable. With the application of stereotactic equipment and anticoagulant urokinase, it is endowed with less invasive damage, higher precision, and a hematoma clearance rate than traditional surgical methods. Conceptually, in addition to reducing the space-occupying effect and intracranial pressure, the evacuation of the hematoma is also aimed at removing the hemorrhagic products that cause acute inflammation and degradation of the brain parenchyma. In the present study, stereotactic aspiration was found to be a strong protective factor for survival but not for consciousness. Hematoma clearance by stereotactic aspiration is effective in removing the lethal factors but does not prevent subsequent edema and arterial necrosis, which usually begin and exacerbate the symptoms 6 h after the initial hemorrhage in the brainstem, which may be the cause of the unimproved consciousness (26).

### 4.5. Indicators for short- and long-term clinical outcomes

In selecting the prognostic indicators, we aimed to cover both the short-term prognosis and the mid-term to long-term prognoses of the patients. According to an epidemiological study based on 20 studies including 1,437 patients, the all-cause 30-day mortality rate among patients with pontine hemorrhage was ~ 48% (6). These results generally seem to represent the natural course of the condition because surgery was seldom performed. Moreover, at our institute, we found that death after 30 days was often not directly caused by the brainstem hemorrhage itself but rather by complications (mainly infection), which led to many confounding factors (nursing level, treatment conditions for subsequent rehabilitation, etc.) that further affected the long-term survival rate. Therefore, the 30-day survival was chosen as a suitable short-term indicator.

Similarly, the 90-day consciousness outcome was selected as a mid-term to long-term prognostic indicator. Patients with PBSH are prone to early coma (90% of patients in this study were in a coma upon admission), and it takes a certain amount of time to become conscious. The reasons for not choosing a longer period of time were the lack of follow-up time for some patients and the smaller difference in the long-term consciousness rate than in the 90-day consciousness rate between groups.

### 4.6. Limitations

The present study had some limitations. First, this retrospective study was performed using data obtained at a single institute, so our results may not accurately represent the national situation. Second, it is debatable to conclude that surgical treatment is the more effective treatment modality in patients with PBSH due to the lack of randomization and the selection bias introduced by choosing treatment modalities. Although our indication for stereotactic aspiration (hematoma volume > 5 ml and GCS <8) was much more stringent than the selection criteria for all 211 included patients, stereotactic aspiration was still found to be a strong protective factor for 30-day survival. Even so, such a selection bias has affected the generalizability of our findings. In the future, a prospective, randomized clinical trial with the involvement of multiple centers and other ethnicities is necessary for better evaluation of the effect of stereotactic aspiration in patients with PBSH. Third, the means of determining clinical outcomes differed from those used in similar studies. Important functional improvement may have been overlooked due to the inclusion of a simple primary outcome score to reduce recall bias.

To overcome these limitations, a more comprehensive, comparative, and prospective study is required, with detailed outcome measures such as functional improvement. Nevertheless, it is hoped that knowledge of the predictors of mortality and functional recovery identified in the present study will improve patient outcomes and individual patient management.

## Data availability statement

The raw data supporting the conclusions of this article will be made available by the authors, without undue reservation.

## Ethics statement

The studies involving human participants were reviewed and approved by the Ethics Committee of the Second Affiliated Hospital Zhejiang University School of Medicine. The patients/participants provided their written informed consent to participate in this study.

## Author contributions

All the authors participated in analyzing and discussing the literature, commenting on, and approving the manuscript. All authors read and approved the final manuscript.
