# Corrigendum: Prognostic models for survival and consciousness in patients with primary brainstem hemorrhage

**DOI:** 10.3389/fneur.2023.1320430

**Published:** 2023-11-06

**Authors:** Jingyi Zhou, Rui Wang, Jizhong Mao, Yichen Gu, Anwen Shao, Fengqiang Liu, Jianmin Zhang

**Affiliations:** ^1^Department of Neurosurgery, Second Affiliated Hospital, School of Medicine, Zhejiang University, Hangzhou, Zhejiang, China; ^2^Liangzhu Laboratory, Zhejiang University Medical Center, Hangzhou, Zhejiang, China; ^3^Brain Research Institute, Zhejiang University, Hangzhou, Zhejiang, China; ^4^Collaborative Innovation Center for Brain Science, Zhejiang University, Hangzhou, Zhejiang, China; ^5^Stroke Research Center for Diagnostic and Therapeutic Technologies of Zhejiang Province, Hangzhou, China; ^6^Clinical Research Center for Neurological Diseases of Zhejiang Province, Hangzhou, China

**Keywords:** primary brainstem hemorrhage, consciousness, multiple logistic regression, predictive factors, stereotactic aspiration

In the published article, there was an error regarding the affiliations for Jizhong Mao. As well as having affiliation 1, they should also have “Liangzhu Laboratory, Zhejiang University Medical Center, Hangzhou, Zhejiang, China”.

The authors apologize for this error and state that this does not change the scientific conclusions of the article in any way. The original article has been updated.

