# Supplementary material for: Prognostic models for survival and consciousness in patients with primary brainstem hemorrhage
Source: Front Neurol. 2023 Feb 23;14:1126585. doi: 10.3389/fneur.2023.1126585 (PMC9995821; doi:10.3389/fneur.2023.1126585)
Supplement: Supplementary file 1 [file Table_1.DOCX]

**Table S1. Associations of Characteristics during** **Hospitalization with 30-day Survival.**

| **Characteristic** | **All patients**  **(n = 211)** | **Survivor**  **(n = 147)** | **Non-survivor**  **(n = 64)** | **P value** | **OR (95% CI)** |
| --- | --- | --- | --- | --- | --- |
| **Laboratory blood examinations during hospitalization** | | | | |  |
| Blood creatinine increase (μmol/L) | 8 ([-2]-[33]) | 6 ([-3]-[27]) | 11.5 (0-76) | 0.064 | 1.00 (1.00-1.00) |
| Blood sodium increase (μmol/L) | 4.8 (1.0-10.7) | 3.9 (0.7-8.7) | 6.9 (2.8-17.7) | 0.002^**^ | 0.95 (0.92-0.98) |
| Blood sodium decrease (μmol/L) | -7.0 ([-12.0]-[-2.9]) | -8.6 ([-13.2]-[-5.0]) | -2.3 ([-6.5]-[0]) | < 0.001^***^ | 0.86 (0.82- 0.92) |
| Blood potassium increase (μmol/L) | 0.9 (0.4-1.4) | 0.9 (0.5-1.3) | 0.7 (0.2-1.5) | 0.536 | 0.79 (0.57-1.09) |
| Blood potassium decrease (μmol/L) | -0.4 ([-0.9]-[-0.1]) | -0.4 ([-0.9]-[ -0.1]) | -0.4 ([-0.9]-[-0.2]) | 0.157 | 1.26 (0.79-2.00) |
| **Treatment method** | | | | |  |
| Stereotactic aspiration | 113 (54%) | 91 (62%) | 22 (34%) | < 0.001^***^ | 3.13 (1.67-5.88) |
| **Complications during hospitalization** | | | | |  |
| High fever | 139 (66%) | 97 (66%) | 42 (66%) | 0.978 | 1.02 (0.55-1.89) |
| Intracranial infection | 3 (1%) | 2 (1%) | 1 (2%) | 0.909 | 0.87 (0.08-10.00) |
| Pneumonia | 185 (88%) | 131 (89%) | 54 (84%) | 0.067 | 1.52 (0.65-3.57) |
| Other infections | 22 (10%) | 17 (12%) | 5 (8%) | 0.565 | 1.54 (0.54-4.35) |
| Deep venous thrombosis | 12 (6%) | 10 (7%) | 2 (3%) | 0.461 | 2.27 (0.48-11.11) |
| Myocardial injury | 28 (13%) | 15 (10%) | 13 (20%) | 0.077 | 0.45 (0.20-1.01) |
| Bed sores | 30 (14%) | 24 (16%) | 6 (9%) | 0.265 | 1.89 (0.73-4.76) |
| Secondary epilepsy | 10 (5%) | 8 (5%) | 2 (3%) | 0.707 | 1.79 (0.37-8.33) |
| Digestive tract hemorrhage | 3 (1%) | 3 (2%) | 0 (0%) | 0.604 | *NA* |
| *P < 0.05, **P < 0.01, ***P < 0.001  Data are expressed as n (%), mean ± SD, median (IQR), OR (95% CI), as appropriate.  The increase was the maximum value of this index during hospitalization minus the value of admission.  The decrease was the minimum value of this index during hospitalization minus the value of admission.  NA, not available. | | | | | |

**Table S2. Associations of Characteristics upon Admission with 90-day Consciousness**

| **Characteristic** | **All patients**  **(n = 208)** | **Conscious patients**  **(n = 67)** | **Unconscious patients**  **(n = 141)** | **P value** | **OR (95% CI)** |
| --- | --- | --- | --- | --- | --- |
| **Clinical characteristics upon admission** | | | | |  |
| Heart rate | 84 (71-101) | 82 (70-96) | 85.5 (71-107) | 0.120 | 0.98 (0.97-1.00) |
| Respiratory rate | 12 (4-15) | 14 (6-16) | 12 (2-15) | 0.039^*^ | 1.04 (1.00-1.09) |
| Systolic blood pressure (mmHg) | 166 ± 31 | 166 ± 29 | 166 ± 32 | 0.985 | 1.00 (0.99-1.02) |
| Diastolic blood pressure (mmHg) | 95 ± 19 | 94 ± 20 | 95 ± 19 | 0.679 | 0.99 (0.97-1.02) |
| Anisocoria | 56 (27%) | 14 (21%) | 42 (30%) | 0.237 | 0.62 (0.31-1.23) |
| Mydriasis | 36 (17%) | 5 (7%) | 31 (22%) | 0.017^*^ | 0.29 (0.11-0.78) |
| Pinpoint pupils | 55 (26%) | 14 (21%) | 41 (29%) | 0.279 | 0.65 (0.32-1.28) |
| Abnormal light reflex | 180 (87%) | 50 (75%) | 130 (92%) | 0.002^**^ | 0.25 (0.11-0.57) |
| GCS score | 4 (3-5) | 5 (3-9) | 3 (3-4) | < 0.001^***^ | 1.40 (1.22-1.61) |
| Emergency hemostatic drugs | 77 (37%) | 29 (43%) | 48 (34%) | 0.302 | 1.47 (0.81-2.70) |
| Emergency mechanical ventilation | 187 (90%) | 55 (82%) | 132 (94%) | 0.020^*^ | 0.31 (0.12-0.78) |
| Emergency decompression | 120 (58%) | 40 (60%) | 80 (57%) | 0.843 | 1.12 (0.63-2.04) |
| Emergency external ventricular drainage | 13 (6%) | 4 (6%) | 9 (6%) | 0.909 | 0.93 (0.28-3.13) |
| **Image characteristics upon admission** | | | | |  |
| Hematoma volume (mL) | 9.6 (5.8-14.8) | 6.3 (3.2-10.4) | 11.6 (7.7-16.2) | < 0.001^***^ | 0.85 (0.80-0.91) |
| Location of hemorrhage | | | | |  |
| Dorsal location | 99 (48%) | 18 (27%) | 81 (57%) | < 0.001^***^ | 0.27 (0.14-0.51) |
| Crossing the midline | 188 (90%) | 55 (82%) | 133 (94%) | 0.011^*^ | 0.28 (0.11-0.71) |
| Midbrain | 150 (72%) | 42 (63%) | 108 (77%) | 0.054 | 0.51 (0.27-0.96) |
| Pons | 204 (98%) | 65 (97%) | 139 (99%) | 0.819 | 0.47 (0.06-3.33) |
| Medulla | 11 (5%) | 2 (3%) | 9 (6%) | 0.306 | 0.45 (0.09-2.13) |
| Extension of hemorrhage | | | | | |
| Cerebellum | 33 (16%) | 8 (12%) | 25 (18%) | 0.387 | 0.63 (0.27-1.47) |
| Thalamus | 24 (12%) | 3 (4%) | 21 (15%) | 0.028^*^ | 0.27 (0.08-0.93) |
| Basal ganglia | 23 (11%) | 3 (4%) | 20 (14%) | 0.037^*^ | 0.28 (0.08-0.99) |
| Ventricle | 124 (60%) | 28 (42%) | 96 (68%) | < 0.001^***^ | 0.34 (0.18-0.61) |
| Hydrocephalus | 8 (4%) | 0 (0%) | 8 (6%) | 0.047^*^ | *NA* |
| **Laboratory blood examinations upon admission** | | | | |  |
| White blood cell count (10^12^/L) | 11.3 (9.0-13.7) | 11.2 (9.3-13.6) | 11.4 (8.9-14.0) | 0.692 | 0.97 (0.91-1.04) |
| Hemoglobin (g/L) | 133.2 ± 20.6 | 131.8 ± 16.9 | 134.4 ± 22.1 | 0.347 | 0.99 (0.98-1.01) |
| Platelet (10^9^/L) | 179.8 ± 60.4 | 181.8 ± 51.7 | 178.4 ± 66.3 | 0.684 | 1.00 (1.00-1.01) |
| C-reactive protein (mg/L) | 31 (10-77) | 28 (11-57) | 33 (10-80) | 0.451 | 1.00 (0.99-1.00) |
| Blood glucose (mmol/L) | 7.6 (6.5-8.9) | 7.5 (6.5-8.5) | 7.8 (6.4-9.3) | 0.361 | 1.02 (0.96-1.10) |
| Blood creatinine (μmol/L) | 76 (62-108) | 69 (58-93) | 81 (65-116) | 0.029^*^ | 0.99 (0.99-1.00) |
| TnT (ng/mL) | 0.02 (0.01-0.05) | 0.01 (0.01-0.02) | 0.03 (0.01-0.06) | < 0.001^***^ | 0.00 (0.00-0.01) |
| APTT (s) | 36.3 ± 6.0 | 34.3 ± 4.1 | 37.2 ± 6.6 | < 0.001^***^ | 0.91 (0.85-0.97) |
| D-Dimer (μg/L) | 2045 (882-4962) | 1830 (850- 3860) | 2240 (890-5040) | 0.342 | 1.00 (1.00-1.00) |
| *P < 0.05, **P < 0.01, ***P < 0.001  Data are expressed as n (%), mean ± SD, median (IQR), OR (95% CI), as appropriate.  NA, not available. | | | | | |
